# Long-term PM2.5 exposure disrupts corneal epithelial homeostasis by impairing limbal stem/progenitor cells in humans and rat models

**DOI:** 10.1186/s12989-023-00540-y

**Published:** 2023-09-27

**Authors:** Shengjie Hao, Zhijian Chen, Yuzhou Gu, Lu Chen, Feiyin Sheng, Yili Xu, Di Wu, Yu Han, Bing Lu, Shuying Chen, Wei Zhao, Houfa Yin, Xiaofeng Wang, S. Amer Riazuddin, Xiaoming Lou, Qiuli Fu, Ke Yao

**Affiliations:** 1 Eye Center of the 2nd Affiliated Hospital, School of Medicine, Zhejiang Provincial Key Lab of Ophthalmology, Zhejiang University, Hangzhou, 310009 Zhejiang Province China; 2 Department of Environmental and Occupational Health, Zhejiang Provincial Center for Disease Control and Prevention, Hangzhou, 310051 Zhejiang Province China; 3 The Wilmer Eye Institute, Johns Hopkins University School of Medicine, Baltimore, USA

**Keywords:** Ambient fine particulate matter, Limbal stem cells, Limbal microenvironments, Circadian rhythm

## Abstract

**Background:**

Limbal stem/progenitor cells (LSPCs) play a crucial role in maintaining corneal health by regulating epithelial homeostasis. Although PM2.5 is associated with the occurrence of several corneal diseases, its effects on LSPCs are not clearly understood.

**Methods:**

In this study, we explored the correlation between PM2.5 exposure and human limbal epithelial thickness measured by Fourier-domain Optical Coherence Tomography in the ophthalmologic clinic. Long- and short-term PM2.5 exposed-rat models were established to investigate the changes in LSPCs and the associated mechanisms.

**Results:**

We found that people living in regions with higher PM2.5 concentrations had thinner limbal epithelium, indicating the loss of LSPCs. In rat models, long-term PM2.5 exposure impairs LSPCs renewal and differentiation, manifesting as corneal epithelial defects and thinner epithelium in the cornea and limbus. However, LSPCs were activated in short-term PM2.5-exposed rat models. RNA sequencing implied that the circadian rhythm in LSPCs was perturbed during PM2.5 exposure. The mRNA level of circadian genes including Per1, Per2, Per3, and Rev-erbα was upregulated in both short- and long-term models, suggesting circadian rhythm was involved in the activation and dysregulation of LSPCs at different stages. PM2.5 also disturbed the limbal microenvironment as evidenced by changes in corneal subbasal nerve fiber density, vascular density and permeability, and immune cell infiltration, which further resulted in the circadian mismatches and dysfunction of LSPCs.

**Conclusion:**

This study systematically demonstrates that PM2.5 impairs LSPCs and their microenvironment. Moreover, we show that circadian misalignment of LSPCs may be a new mechanism by which PM2.5 induces corneal diseases. Therapeutic options that target circadian rhythm may be viable options for improving LSPC functions and alleviating various PM2.5-associated corneal diseases.

**Supplementary Information:**

The online version contains supplementary material available at 10.1186/s12989-023-00540-y.

## Background

Corneal epithelium on the surface of the cornea acts as a natural barrier and prevents the ocular surface from environmental stimuli [1]. Corneal epithelial homeostasis largely regulates corneal health, its disruption can lead to serious corneal damage and multiple corneal diseases [2, 3].

Limbal stem/progenitor cells (LSPCs), located in the corneoscleral transition zone, are the primary source of corneal epithelial cells, essential for maintaining corneal epithelial homeostasis [4]. LPSCs are resident in a unique cellular niche that contains blood vessels, nerves, lymphatic vessels, stromal cells, melanocytes, immune cells, extracellular matrix (ECM), and soluble growth factors, all of which constitute the limbal microenvironment [5]. The niche microenvironment is necessary for the survival and functions of LPSCs, particularly for their stemness [6]. Dysfunction of LSPCs or destruction of their microenvironment will lead to LSPCs damage and eventually to limbal stem cell deficiency (LSCD), which manifests as thinner limbal epithelial thickness, impaired epithelial wound healing, and opacification [7], and results in blindness in severe cases [8, 9].

PM2.5, one of the main hazardous components of air pollution, specifically referring to fine particulate matter with a diameter less than 2.5 microns, has been previously linked to ocular surface diseases such as dry eye, pterygium, and conjunctivitis [10–12]. We previously pointed out that autophagy is one of the main mechanisms of corneal damage after PM2.5 exposure [13, 14]. However, the specific effect of PM2.5 on corneal epithelial homeostasis, which is related to LSPCs and their microenvironment, has yet to be fully established.

Recently, a few studies have shown that PM2.5 exposure disrupts circadian rhythm and causes functional or metabolic disorders in different organs [15, 16]. Circadian rhythm refers to the regular variation of life activities in 24-hour cycles, which controls multiple physiological processes and maintains internal homeostasis based on light/dark cycles, sleep/wake rhythms, or feeding patterns [17]. This system is regulated by a transcriptional translation feedback loop composed of several core clock genes (Bmal1, Clock, Period (Per1, Per2, and Per3), Cryptochrome (Cry1 and Cry2), Rev-erbα and Rorα) [17]. Studies have shown that light pollution or sleep loss-induced circadian rhythm disturbance can cause damage to the ocular tissues, such as the retina and lacrimal glands [18, 19]. We hypothesize that PM2.5-induced damage of corneal epithelial homeostasis may be mediated by alteration of the circadian rhythm of LSPCs.

In this study, we analyzed the correlation between PM2.5 exposure and the limbal impairment in humans, explored changes in LSPCs and their microenvironment using PM2.5-exposure rat models, and investigated the role of circadian rhythm in LSPCs under PM2.5 exposure.

## Results

### Correlation between PM2.5 and limbal epithelium thickness in humans

**Fig. 1:**
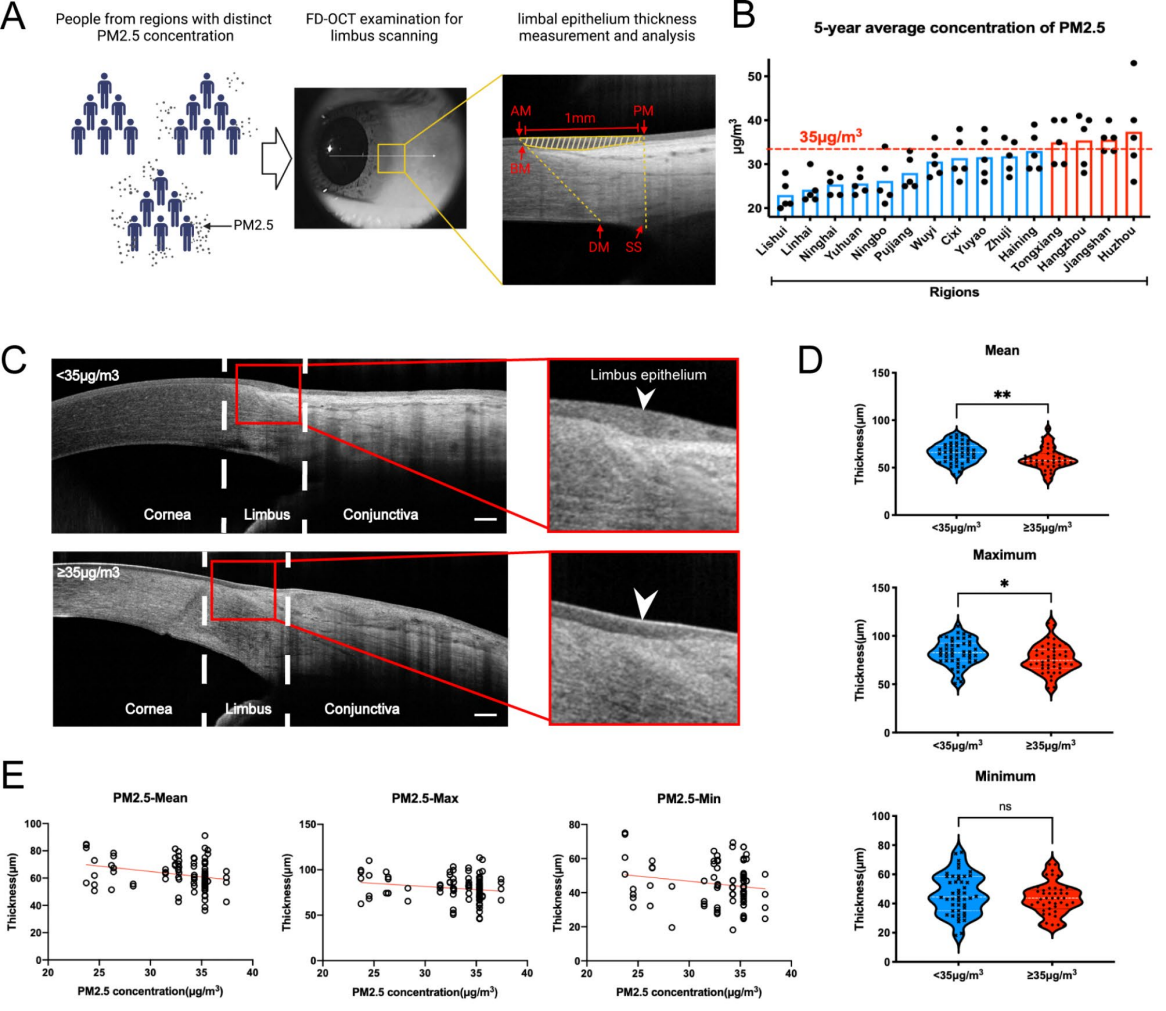
Correlation between PM2.5 and limbal epithelium thickness in humans. (**A**) FD-OCT images of people from regions with a distinct annual average concentration of PM2.5 were collected and limbal epithelium thickness was measured using IPP6.0. The limbal epithelium scale was identified according to its anatomical marks. Shaded areas indicated the target limbal epithelium. AM, anterior margin of limbus; PM, posterior margin of limbus; SS, scleral spur; BM, Bowman’s membrane; DM, Descemet’ s membrane. **(B)** The 5-year average concentration of PM2.5 in people from different regions in Zhejiang Province. Every plot indicated an annual average concentration of PM2.5 (2018–2022). Data were publicly released by the Ecological Environment Department of Zhejiang. **(C)** FD-OCT image of the limbus in two groups (scale bar, 250 μm). White arrowhead indicated limbal epithelium. **(D)** Violin plot diagram of mean/maximum/minimum value in two groups (*p < 0.05, **p < 0.01). **(E)** Spearman analysis was conducted to separately define the correlation between PM2.5 concentration and mean/maximum/minimum value of limbal epithelium thickness. The red line indicates the fitted regression line. FD-OCT: Fourier-domain Optical Coherence Tomography

As depicted in the meta-analysis (Fig. S1), there is a clear positive association between exposure to fine particulate matter and the occurrence of corneal disease or related symptoms. These findings suggested that disruptions in corneal epithelial homeostasis may serve as the underlying cause of various corneal diseases. Corneal epithelial homeostasis is primarily maintained by LSPCs. Here we investigated the effects of PM2.5 on LSPCs by measuring the limbal epithelium thickness of people from different regions with different air qualities in Zhejiang Province (Fig. 1A).

A total of 89 individuals were included in the present study based on the set inclusion and exclusion criteria. The participants included 47 males and 42 females. A total of 28 of them were above 60 years of age, 53 were between 35 and 59 old, and 8 were between 18 and 35 old (Table 1). According to Air Quality Standards (GB3095-2012), patients from different regions of Zhejiang Province were divided into 2 groups based on the 5-year average PM2.5 concentration: <35 µg/m^3^ and ≥ 35 µg/m^3^**(**Fig. 1B**)**. There was no significant difference in age and gender ratio between individuals in the two groups (Table 1).

**Table 1:** Demographic characteristics of people subjects

	5-year average PM2.5 concentration					
	< 35 µg/m^3^		≥ 35 µg/m^3^		Total	Analysis
Characteristic						
	N	%	N	%	N	*p*
Gender						
Male	24	52.17	23	53.49	47	0.497
Female	22	47.83	20	46.51	42	
Age						
18–35	4	8.70	4	9.30	8	0.901
35–59	30	65.22	23	53.49	53	
≥ 60	12	26.09	16	37.21	28	
Total	46	100.00	43	100.00	89	

Results of this study found that the people from regions with PM2.5 concentration above 35 µg/m^3^ had a thinner limbal epithelium (Fig. 1C). This was evidenced by statistically lower mean and maximum values (*P* < 0.05) in this group (regions with PM2.5 concentration above 35 µg/m^3^) as compared with that in the other group (regions with PM2.5 concentration below 35 µg/m^3^), while there was no significant change in minimum values (*P* > 0.05; Fig. 1D, Table S1). The results showed that long-term exposure to PM2.5 exceeding 35 µg/m^3^ may cause the loss of LSPCs.

Spearman correlation analysis was used to assess the correlation between PM2.5 concentration and limbal epithelial thickness. The data obtained in this study showed that the mean, maximum, and minimum corneal limbal epithelial thicknesses decreased with an increase in PM2.5 concentration (Fig. 1E). It was particularly found that there was a statistical difference in the negative correlation (correlation coefficient = -0.2667) between the mean value and PM2.5 concentration (*P* = 0.0115). Further, the rate of thinning was − 0.7875 μm/(µg•m^− 3^), which indicated that for every 1 µg/m^3^ increase in PM2.5 concentration, the average thickness of corneal limbal epithelium decreased by 0.7875 μm (Table S2). All of these results suggested that long-term exposure to PM2.5 may cause the loss of LSPCs in the population and increase the risk of developing LSCD, which further affected corneal epithelium homeostasis.

### Long-term PM2.5 exposure breaks corneal epithelial homeostasis of rats

**Fig. 2:**
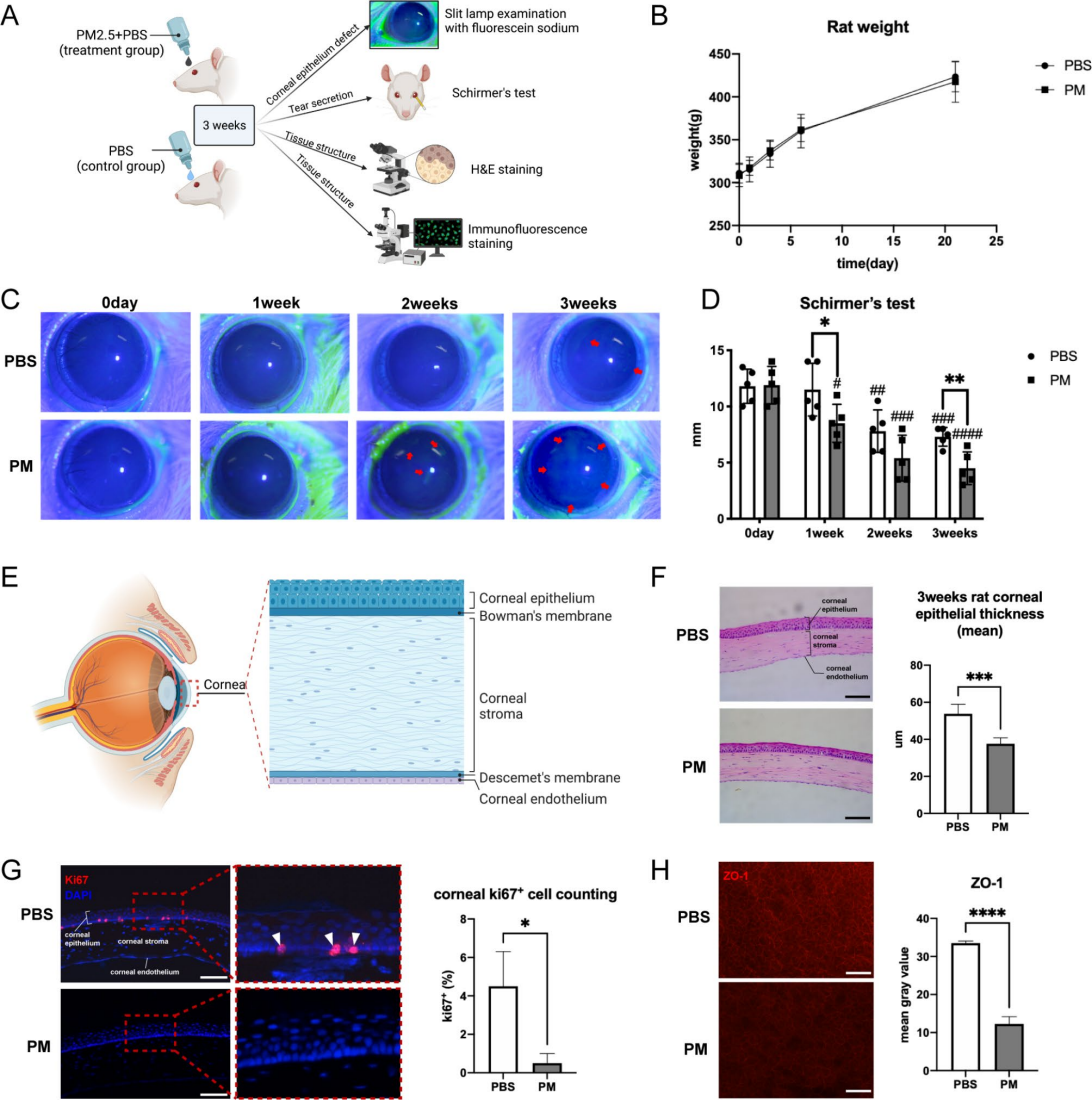
Long-term chronic exposure to PM2.5 breaks corneal homeostasis of rats. (**A**) A long-term PM2.5-exposed rat model was established, and ocular surface health was monitored by ophthalmological (slit lamp examination and Schirmer’s test), pathological (H&E staining), and molecular biological technologies (immunofluorescence staining). **(B)** Rat body weights of the PM2.5 or PBS group in 3 weeks. N = 5 in each group. **(C)** Slit lamp examination was used to examine corneal epithelium defect and results showed more corneal staining of fluorescein sodium occurred under cobalt-blue light in the PM2.5 treatment group. The red arrows pointed to the corneal epithelium defect. N = 5 in each group. **(D)** Schirmer’s test indicated that prolonged PM2.5 exposure caused more severe damage in tear secretion in the PM2.5 group. N = 5 in each group. *p < 0.05, ** *p* < 0.01 compared with indicated group, #*p* < 0.05, ## *p* < 0.01, ### *p* < 0.001, #### *p* < 0.0001 compared with the results of their own group at 0 days. **(E)** Anatomical structures of the cornea. **(F)** H&E staining showed structural changes between the two groups (scale bar, 100 μm). The cornea epithelium thickness of the PM2.5 group got thinner. N = 5 in each group. ***p < 0.001. **(G)** Immunofluorescence staining showed the expression of proliferation marker Ki67 significantly decreased on rat cornea in the PM2.5 group (scale bar, 100 μm). N = 3 in each group. The white arrowhead indicated Ki67^+^ cells. *p < 0.05. **(H)** Immunofluorescence staining showed the expression of tight junction marker ZO-1 decreased on rat cornea in the PM2.5 group (scale bar, 100 μm). N = 3 in each group. ****p < 0.0001. PBS: the PBS eyedrop administration group; PM: the PM2.5 eyedrop administration group

To investigate the precise impact of PM2.5 on corneal epithelial homeostasis, we established a 3-week long-term PM2.5-exposed rat model in this study. Ocular surface health was comprehensively evaluated using ophthalmological, pathological, and molecular biological techniques (Fig. 2A). There was no significant difference in body weight between the two groups of rats during exposure (Fig. 2B).

The ocular surface damage was evaluated by slit lamp examination and Schirmer’s test. Slit lamp examination showed that there was an occurrence of more corneal staining of fluorescein sodium under cobalt-blue light in the PM2.5 treatment group. This meant its corneal epithelium defect was more serious as compared with that in the control group (PBS), and the ocular surface damage worsened with increasing exposure time (Fig. 2C). Schirmer’s test indicated that prolonged PM2.5 exposure caused a more severe reduction in tear secretion compared to the control group (Fig. 2D).

The changes in corneal epithelium barrier and repair functions were evaluated by H&E staining and immunofluorescence staining. Cornea epithelium anatomically lying on the corneal stroma consists of several layers of corneal epithelial cells (Fig. 2E). The thickness of the corneal epithelium of individuals in the PM2.5 group was thinner (Fig. 2F) as compared with that from the control group. The percentage of Ki67^+^ cells in corneal epithelium was lower in the PM2.5 group (Fig. 2G), indicating impaired cell proliferation. Reduced tight junctions were also observed after long-term PM2.5 exposure as showed by staining of ZO-1(Fig. 2H). Therefore, our findings clearly demonstrated that prolonged exposure to PM2.5 in rats resulted in the impairment of corneal barrier function and diminished renewal potential. Consequently, PM2.5 exposure disrupted corneal epithelial homeostasis.

### Long-term PM2.5 exposure damages LSPCs proliferation and differentiation abilities

**Fig. 3:**
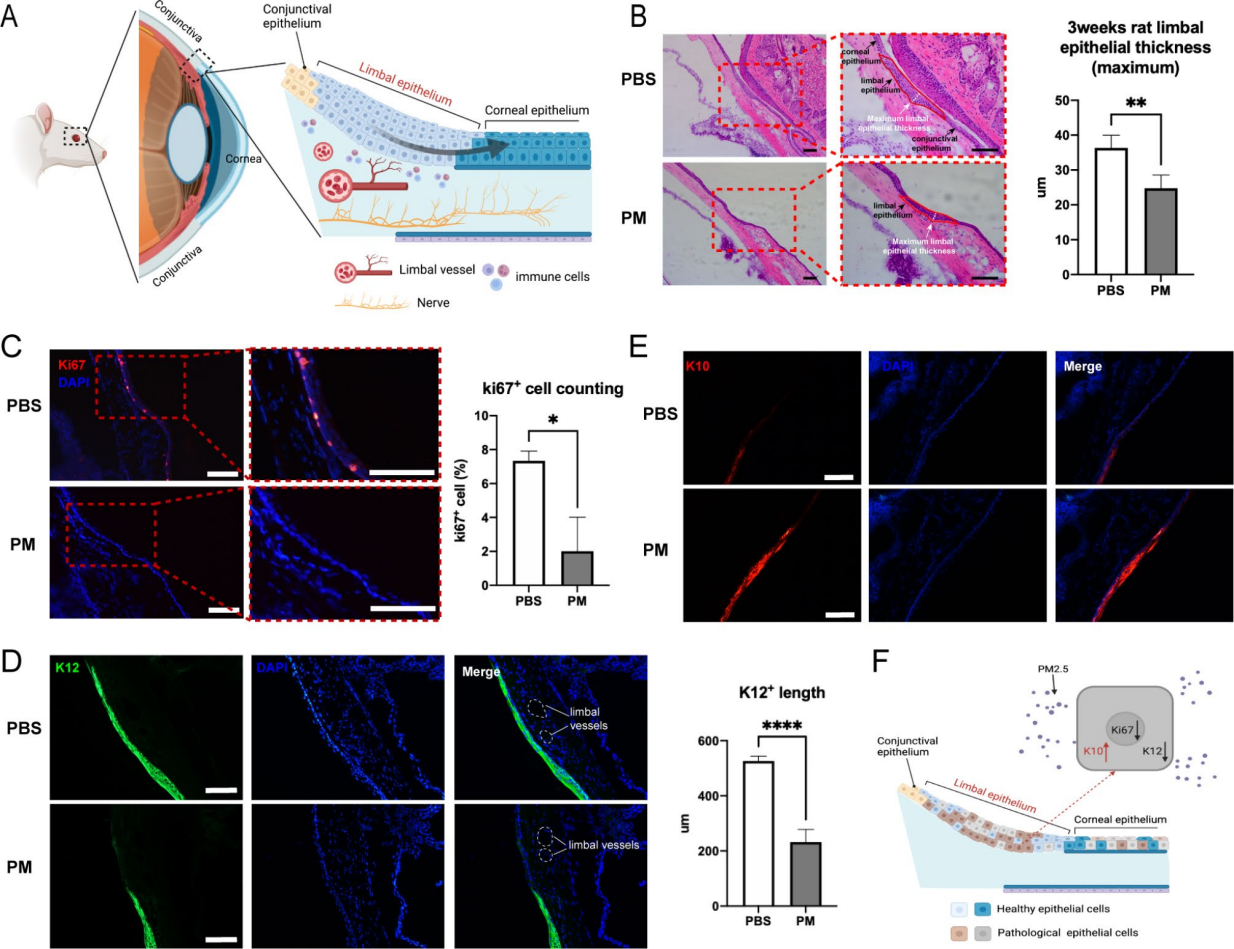
Long-term chronic exposure to PM2.5 damages LSPCs proliferation and differentiation abilities. (**A**) Anatomical structures of the limbus. **(B)** H&E staining showed thinner limbal epithelium suffered by long-term PM2.5 exposure on rat eyes (scale bar, 100 μm). N = 5 in each group. **p < 0.01. **(C)** Immunofluorescence staining showed the expression of proliferation marker Ki67 significantly decreased on rat limbus in the PM2.5 group (scale bar, 100 μm). N = 3 in each group. *p < 0.05. **(D)** K12 was markedly diminished in the PM2.5 group, which meant LSPCs differentiation was inhibited by PM2.5 (scale bar, 100 μm). N = 5 in each group. ****p < 0.0001. **(E)** PM2.5 group showed more K10 expression on the limbus, which implied limbus may have undergone abnormal differentiation under long-term PM2.5 stimulation (scale bar, 100 μm). N = 4 in each group. **(F)** The downregulated expression of Ki67, and K12 and abnormal expression of K10 indicated a loss of normal proliferation and differentiation function in LSPCs, which could lead to disruption of corneal epithelium homeostasis. PBS: the PBS eyedrop administration group; PM: the PM2.5 eyedrop administration group; LSPCs: Limbal stem/progenitor cells; K12: Keratin12; K10: Keratin10

To maintain corneal epithelial homeostasis, LSPCs located in the limbal epithelium have the potential of proliferating and differentiating into corneal epithelium to support its renewal as well as post-damage repair (Fig. 3A). We further investigated the effects of PM2.5 on LSPCs.

Results of H&E staining showed that treatment with PM2.5 caused a decrease in maximum limbal epithelial thickness (Fig. 3B), which is consistent with the results in humans. The percentage of ki67^+^ cells in limbal epithelium also decreased significantly (Fig. 3C), suggesting the potential of the proliferation of the LSPCs was inhibited by long-term exposure to PM2.5. The expression of differentiation marker Keratin12 (K12) on the limbus also decreased, indicating that the differentiation of LSPCs was diminished by PM2.5 (Fig. 3D).

In addition, we also found a strong expression of Keratin10 (K10) in the PM2.5 group, which is generally expressed in epidermis-related tissues (Fig. 3E), suggesting that the limbus may have undergone abnormal differentiation under long-term stimulation by PM2.5. Overall, PM2.5 significantly impaired the ability of LSPCs to proliferate and differentiate, and homeostasis of the corneal epithelium could no longer be maintained (Fig. 3F).

### PM2.5 triggers activation of the LSPCs at the early exposure phase

**Fig. 4:**
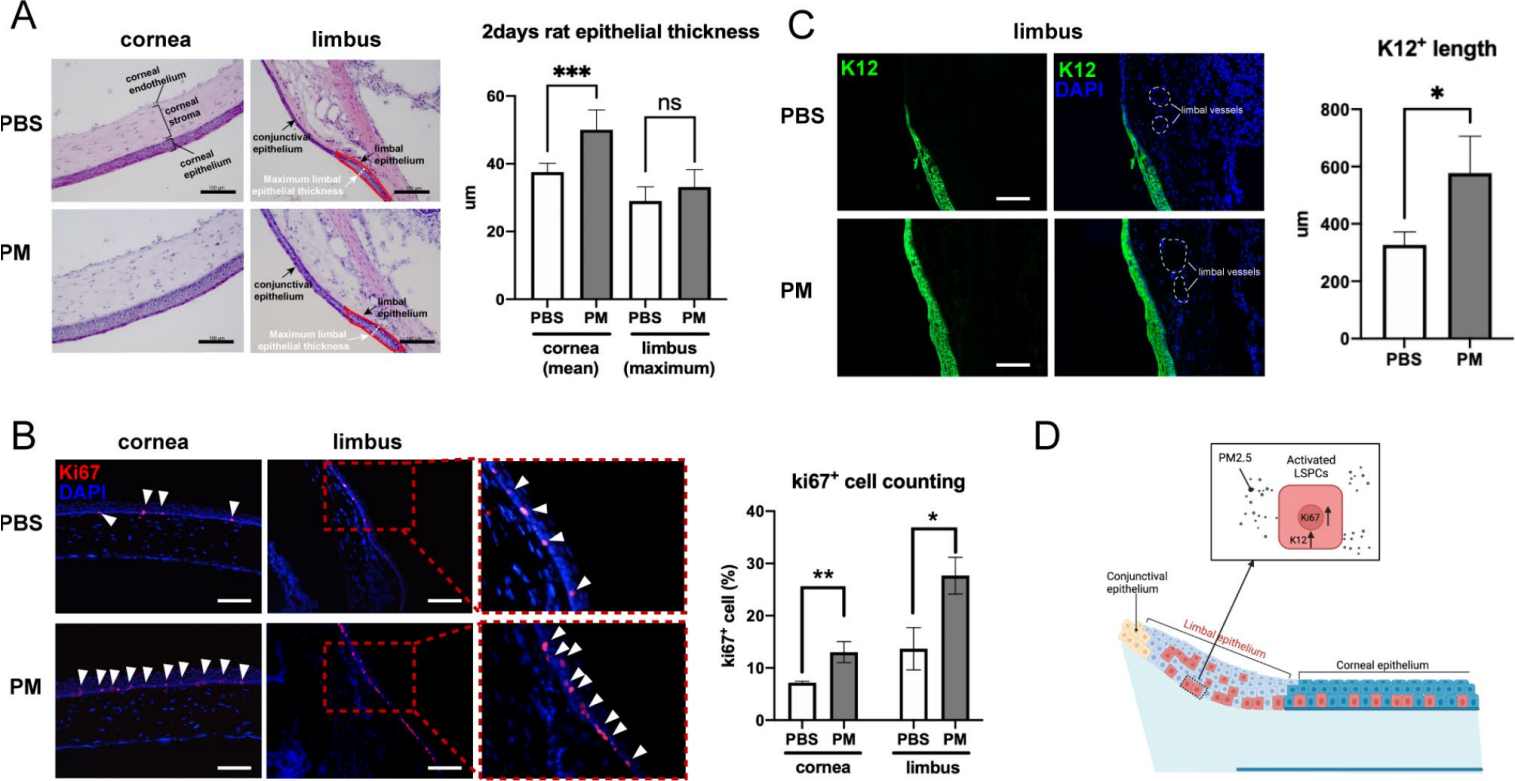
PM2.5 triggers activation of the LSPCs at the early exposure stage. (**A**) H&E staining showed PM2.5 led to corneal epithelium thickening after 2 days of exposure. N = 6 in each group. No significant changes were observed in limbal epithelial thickness. N = 6 in each group. ***p < 0.001. **(B)** Ki67^+^ cells increased on rat corneal and limbal epithelial after exposure to PM2.5 for 2 days (scale bar, 100 μm). N = 3 in each group. White arrowhead points to the Ki67^+^ cells. *p < 0.05, **p < 0.01. **(C)** K12 significantly increased on rat limbus after PM2.5 exposure for 2 days, suggesting active differentiation was induced at an early stage (scale bar, 100 μm). N = 4 in each group. *p < 0.05. **(D)** PM2.5 triggers the activation of the LSPCs at the early exposure stage and increases the proliferation and differentiation of LSPCs. PBS: the PBS eyedrop administration group; PM: the PM2.5 eyedrop administration group; LSPCs: Limbal stem/progenitor cells; K12: Keratin12

The changes in corneal epithelial homeostasis and LSPCs triggered by PM2.5 at an early stage were also detected. After 2-day short-term exposure, slit lamp examination showed that there was no significant difference between the PM2.5 treatment group and the control group (Fig. S2), and tear secretion was not notably affected by PM2.5 exposure as well (Fig. S3). While the rats in the PM2.5 group had thickened corneal epithelium and an increased percentage of Ki67^+^ cells (Fig. 4A and B). Furthermore, the proliferation marker ki67 and differentiation marker K12 were also notably expressed at limbal epithelium in the PM2.5 group (Fig. 4A C), pointing out that LSPCs were initiated to proliferate and differentiate toward the corneal epithelium at early PM2.5 exposure to maintain corneal epithelial homeostasis (Fig. 4D).

### Transcriptome analysis of LSPCs under short-term PMexposure

**Fig. 5:**
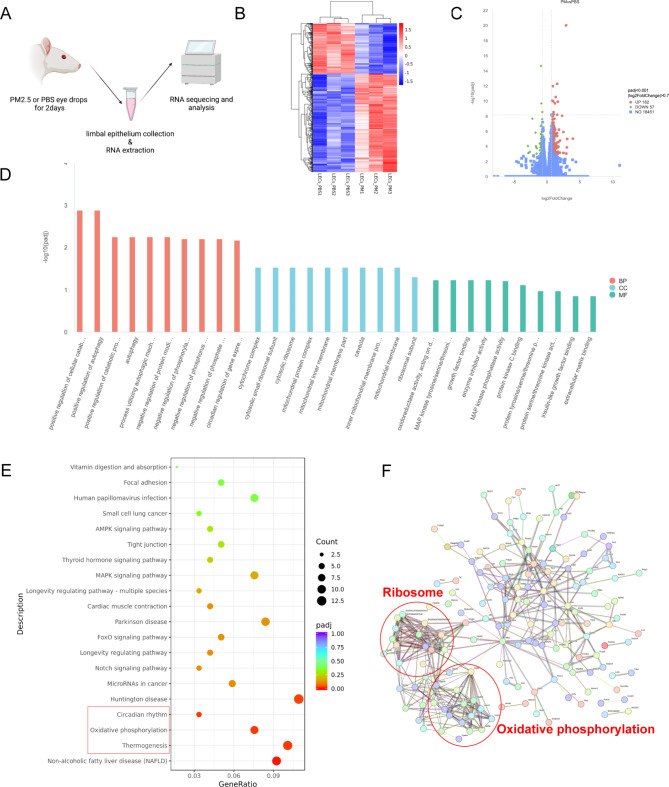
Transcriptome analysis of LSPCs under short-term PM2.5 exposure (**A**) Limbal epithelium mRNA of rats was extracted and RNA-seq was performed. N = 3 in each group. **(B)** Heatmap showed the different expression profiling between PBS and PM2.5 groups. **(C)** The volcano plot indicated the DEGs between PBS and PM2.5 groups. **(D)** GO analysis of DEGs including biological process, cellular component, and molecular function. **(E)** KEGG pathway enrichment of DEGs. **(F)** PPI network analysis of DEGs. LECs_PBS: limbal epithelial cells in the PBS eyedrop administration group; LECs_PM: limbal epithelial cells in the PM2.5 eyedrop administration group; LSPCs: Limbal stem/progenitor cells; RNA-seq: RNA-sequencing; GO: Gene Ontology; KEGG: Kyoto encyclopedia of genes and genomes; DEGs: Differentially expressed genes; PPI: Protein-protein interaction

For exploring the mechanism of PM2.5 affecting LSPCs, transcriptomic profiling of rats limbal epithelium with short-term exposure was performed by RNA-sequencing (RNA-seq) (Fig. 5A). Heat map and the volcano plot were illustrated in Fig. 5B C. In total, 182 significantly up-regulated genes and 57 significantly down-regulated genes were detected (| log2(fold change) | > 0.7, adjusted P value < 0.001). Gene Ontology (GO) enrichment analysis, Kyoto encyclopedia of genes and genomes (KEGG) pathways, and protein-protein interaction (PPI) network analysis were performed based on 239 differentially expressed genes (DEGs). Figure 5D presented the top 10 GO categories for biological process (BP), molecular function (MF), and cellular component (CC). Positive regulation of cellular catabolic process and autophagy were mentioned in BP, and the component of ribosome and mitochondria were mentioned in CC. KEGG pathway analysis indicated that circadian rhythm, thermogenesis, and oxidative phosphorylation pathway were significantly perturbed (Fig. 5E). Oxidative phosphorylation and ribosome-associated clusters were also identified in PPI network analysis (Fig. 5F).

### The activation and dysregulation of LSPCs triggered by PM2.5 exposure is associated with circadian disturbance

**Fig. 6:**
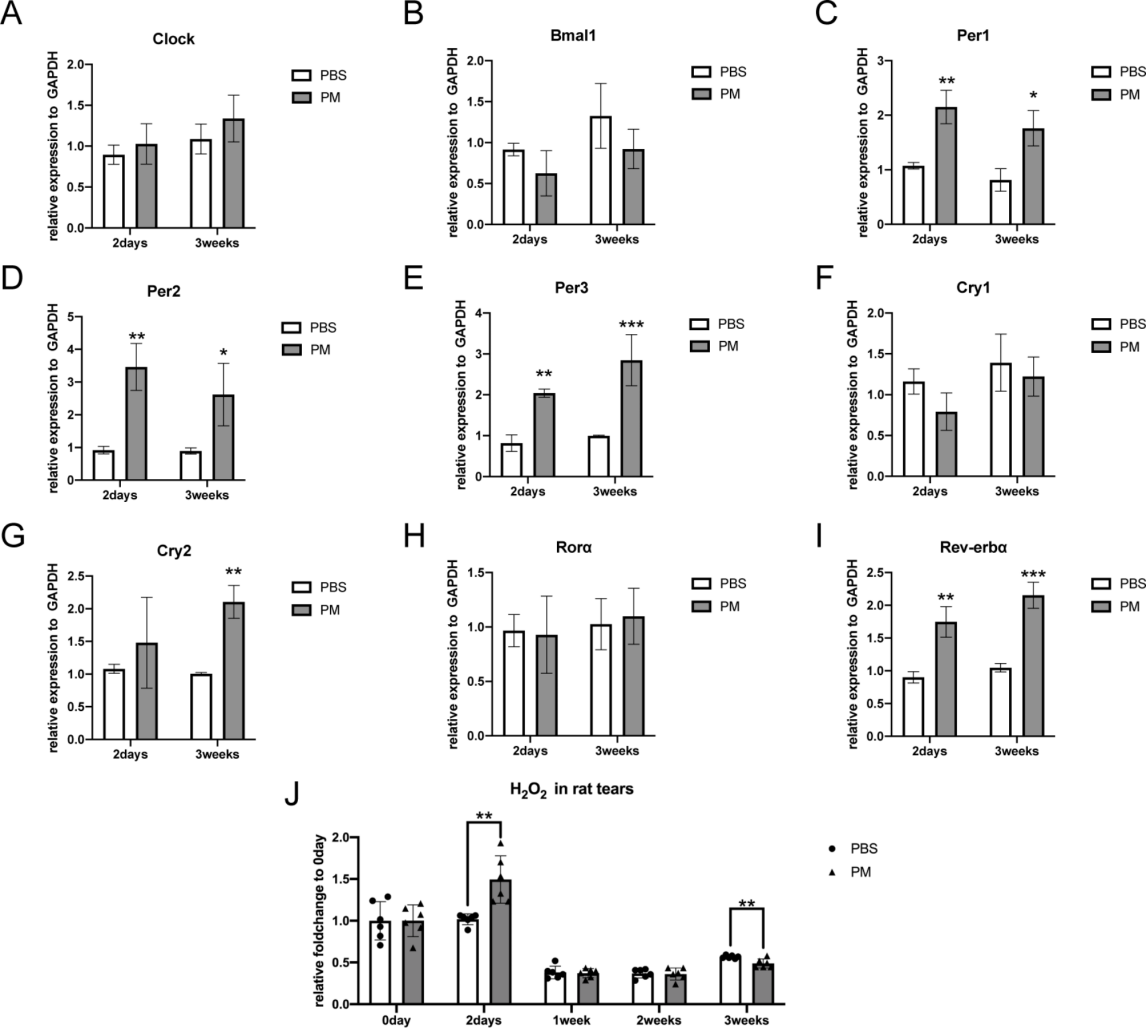
The circadian rhythm was involved in the activation and dysregulation of LSPCs. (**A-I**) The expression of circadian rhythm-related genes in PBS and PM2.5 group at 2 days or 3 weeks. N = 3 in each group. *p < 0.05, **p < 0.01, ***p < 0.001. **(J)** H_2_O_2_ in rat tears was elevated at 2 days in the PM2.5 group and was lower than the control group after 3 weeks. N = 6 in each group. **p < 0.01. PBS: the PBS eyedrop administration group; PM: the PM2.5 eyedrop administration group; LSPCs: Limbal stem/progenitor cells; H_2_O_2_: hydrogen peroxide

Recently, some researchers identified that circadian misalignment could play an important role in PM2.5-induced damage [15, 16]. Therefore, we subsequently focused on the genes involved in the circadian rhythm system. In DEGs, circadian genes including Per1, Per2, and Per3 were highly expressed in the PM2.5 group, with increases of 1.84, 2.89, and 1.90 times, respectively. The mRNA level of core clock genes (Clock, Bmal1, Per1, Per2, Per3, Cry1, Cry2, Rorα, and Rev-erbα) in the short-term exposure model was detected by quantitative fluorescent polymerase chain reaction (qPCR). Expression of Per1, Per2, Per3, and Rev-erbα significantly increased, other genes related to circadian rhythm including Clock, Bmal1, Cry1, Cry2, and Rorα didn’t change obviously (Fig. 6A and I).

To explore the effects of PM2.5 on circadian rhythm in the long term, we further detected the expression of core clock genes in the 3-week exposure model. Results showed that in addition to the gene that elevated in the short-term exposure model, Cry2 also had an obvious increase (Fig. 6A and I). Our results revealed that circadian rhythm was disturbed throughout PM2.5 exposure. Circadian pathways might play different roles in the initiation and damage of LSPCs. Short-term circadian misalignment might contribute to the activation of LSPCs, while long-term circadian rhythm disturbance led to the dysfunction of LSPCs and caused corneal epithelial homeostasis disruption.

Besides, oxidative phosphorylation has also been suggested to be an important factor contributing to PM2.5-induced diseases [20–22]. We examined the changes in hydrogen peroxide (H_2_O_2_) in rat tears throughout the PM2.5 exposure period. Interestingly, H_2_O_2_ in tears was elevated after 2 days of PM2.5 exposure but was not significantly different from control after 1 week and was even lower than control after 3 weeks (**Fig. 6J**), suggesting that oxidative phosphorylation may be involved in the activation of LSPCs, but it may not be the most critical factor in the dysfunction of LSPCs and disruption of corneal homeostasis.

### PM2.5 exposure alters the limbal microenvironment

The limbal microenvironment plays a critical role in providing a specialized stem cell niche for LSPCs and supporting their functions. However, the association between circadian disruption, LSPC dysfunction, and the impact on the limbal microenvironment following PM2.5 exposure remains unclear. Therefore, the present study also investigated the responses of the limbal microenvironment including the corneal neural network, limbal vasculature, and immune cell infiltration in short- or long-term exposure to PM2.5, respectively.

**Fig. 7:**
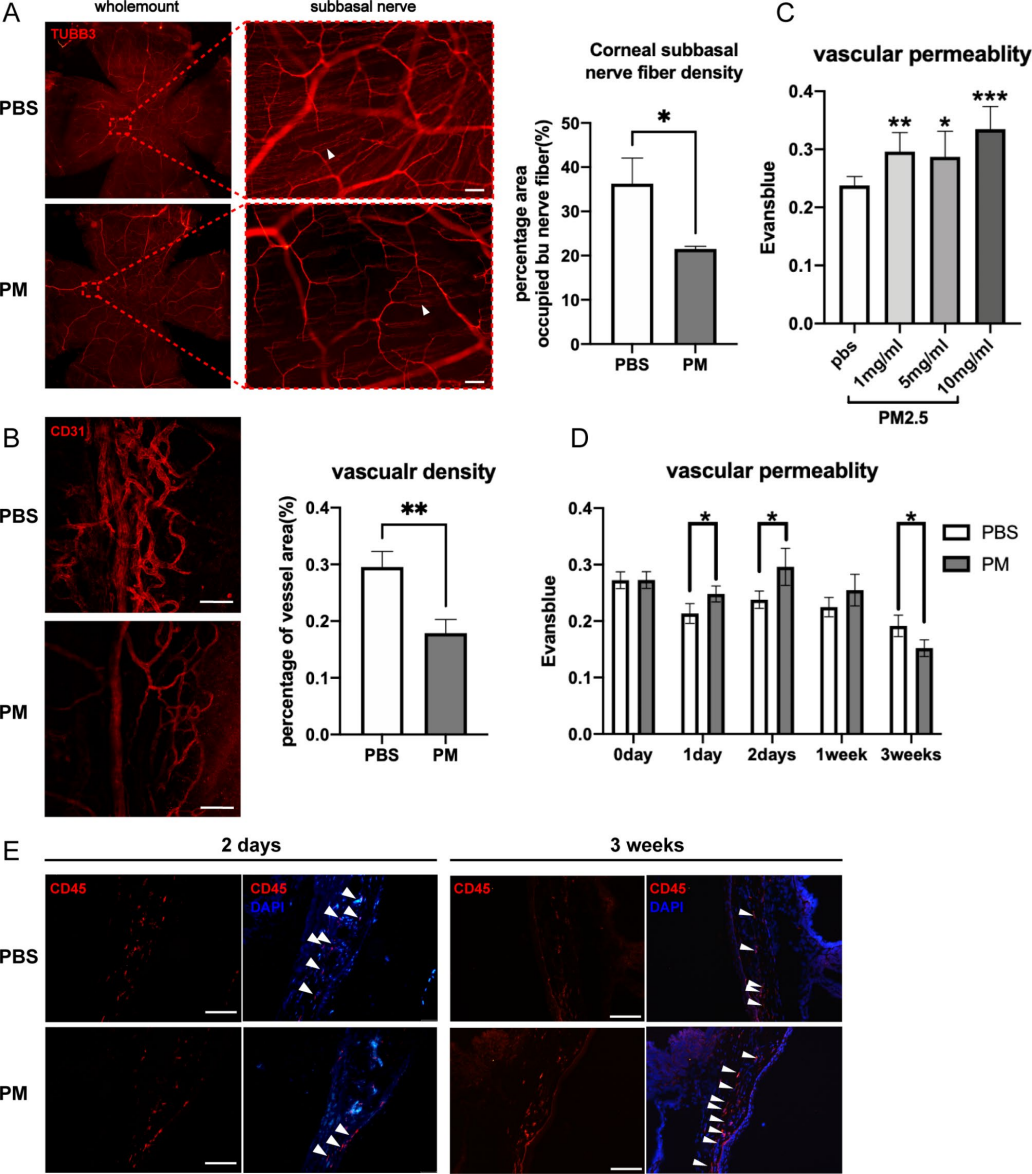
PM2.5 exposure alters the limbal microenvironment. (**A**) Corneal whole mount immunofluorescence staining of TUBB3 showed the morphology change of corneal nerve fiber after exposure for 3 weeks. PM2.5 group showed a decreased density of sub-basal nerve fibers (scale bar, 100 μm). N = 3 in each group. *p < 0.05. **(B)** The change in limbal vessel density at 3 weeks was shown according to immunofluorescence staining of CD31 (scale bar, 100 μm). N = 3 in each group. *p < 0.05. **(C)** Limbal vascular permeability increased in a dose-dependent manner after 2 days of PM2.5 exposure. N = 6 in PBS, 1 mg/ml and 5 mg/ml group. N = 4 in 10 mg/ml group. *p < 0.05, **p < 0.01, ***p < 0.001. **(D)** Limbal vascular permeability in 3 weeks. *p < 0.05. N = 6 in 0, 1, 2 days and 1 week groups. N = 4 in 3 weeks groups. **(E)** In the PM2.5 group, CD45^+^ staining of limbal interstitial cells decreased at 2 days and increased at 3 weeks (scale bar, 100 μm). The white arrowhead indicated the CD45^+^ cells. N = 3 in each group. PBS: the PBS eyedrop administration group; PM: the PM2.5 eyedrop administration group

The immunofluorescence staining of TUBB3 showed that long-term exposure to PM2.5 significantly decreased the density of subbasal nerve fibers (Fig. 7A). Limbal vessels of the rats in the PM2.5 group exhibited a decrease in vascular density by staining for CD31 (Fig. 7B). While no significant alternation in the morphology of vessel and corneal nerves was observed at the early phase (Fig. S4–S5).

In addition, the EvansBlue leakage assay was performed to evaluate limbal vascular permeability. The vascular leakage increased in a dose-dependent manner after 2-day PM2.5 exposure (Fig. 7C). However, when exposed for 3 weeks, vascular permeability was significantly reduced (Fig. 7D), which may be related to the lower vessel density.

**Fig. 8:**
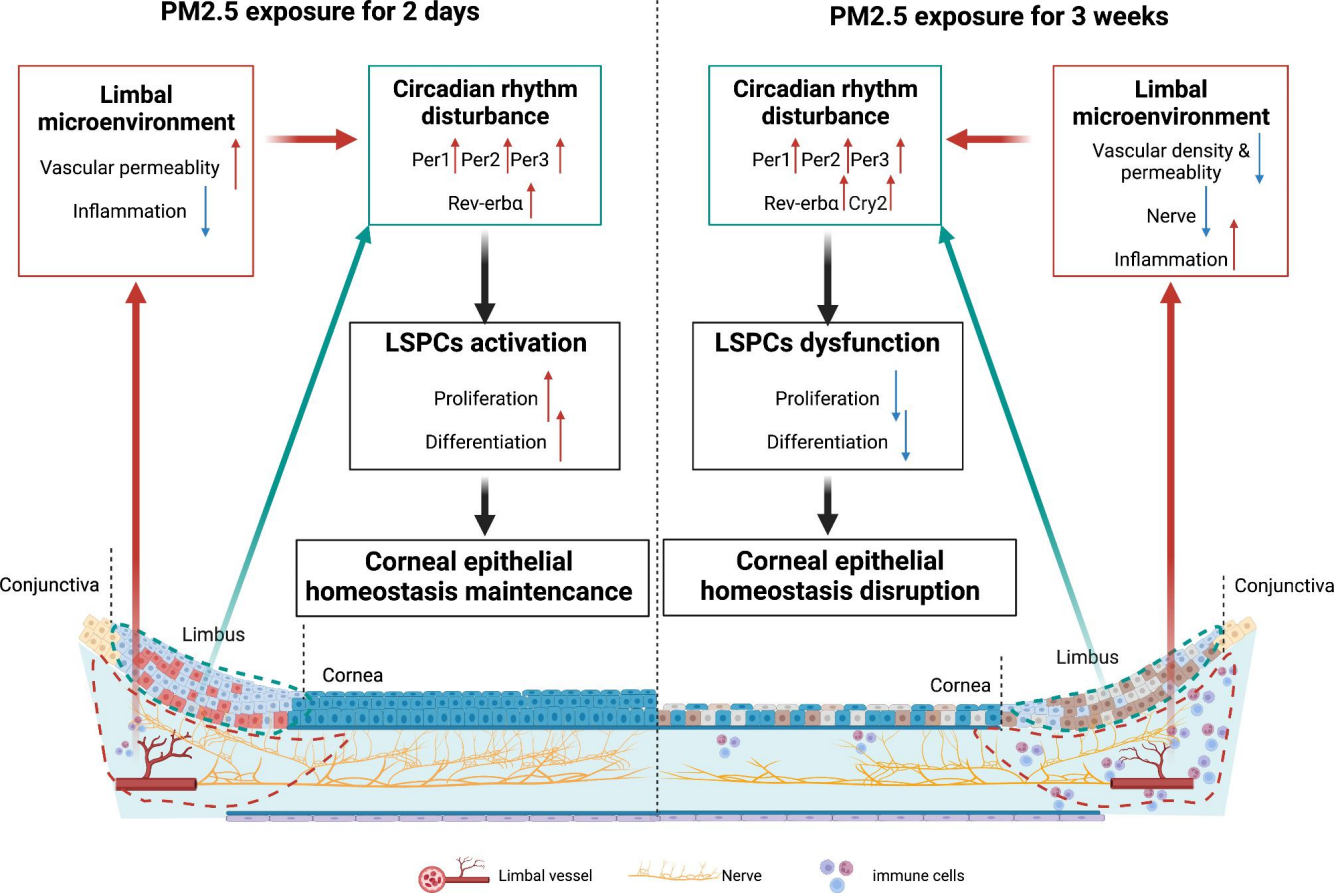
Mechanism overview: Long-term PM2.5 exposure impairs limbal stem/progenitor cells by disrupting the circadian rhythm and their stem cell niche. Long-term PM2.5 exposure destructs the limbal microenvironment manifested as decreased vascular density and permeability, decreased corneal subbasal nerve fiber, and enhanced inflammation, which perturbs the circadian rhythm of LSPCs and causes LSPCs dysregulation. Corneal epithelial homeostasis can no longer be maintained, and a variety of corneal diseases ultimately occur. In addition, short-term microenvironmental responses might be involved in the activation of LSPCs by circadian perturbations to promote the maintenance of corneal epithelial homeostasis. LSPCs: Limbal stem/progenitor cells

Results showed that rats exposed to PM2.5 for 3 weeks possessed a large proportion of CD45^+^ cells that were located in the limbal stroma (Fig. 7E), implying an active inflammation response occurred after exposure to PM2.5. While the number of the immunocyte decreased compared to the control group in the early phase. Overall, short-term PM2.5 exposure disturbed the limbal microenvironment, resulting in circadian misalignment and activation of LSPCs, whereas long-term PM2.5 exposure eventually disrupted stem cell niches and caused LSPCs dysregulation (Fig. 8).

## Discussion

Fine particulate matter has been recognized as a risk factor for multiple corneal diseases [23]. Many studies have focused on the damage and mechanism of PM2.5 directly acting on corneal epithelium [13, 22, 24], and our work, for the first time, elucidates that PM2.5 breaks corneal epithelial homeostasis by damaging LSPCs in humans and animal models, which identified another critical mechanism in air pollution-induced corneal diseases.

### PM2.5 exposure and LSCD

The present findings demonstrated that limbal epithelium thickness was negatively correlated with PM2.5 concentration in humans, and long-term PM2.5 exposure induced mild stage manifestation of LSCD in the rat model, as evidenced by thinner limbal epithelium, corneal epithelial defects, and reduced subbasal nerve density. The etiology of LSCD can be primary, such as aniridia and dyskeratosis congenita [25, 26], or secondary, from external factors that directly damage LSPCs or stem cell niches, such as chemical or thermal injury [27, 28], microbial infections [29], and Stevens-Johnson syndrome [30]. Yang et al. noted that nasal and temporal limbal epithelium becomes thinner with age [31]. Our work here implied that PM2.5 possibly can be another factor that increased the risks of LSCD, and our model could be a novel and effective model for studying the pathogenesis and treatment of LSCD.

### The effects of PM2.5 exposure on LSPCs activity and corneal epithelial homeostasis

LSPCs responded differently to short-term and long-term PM2.5 exposure, resulting in different degrees of corneal damage. LSPCs proliferate and migrate toward the center to repopulate the corneal epithelium, which plays an important role in the maintenance of corneal homeostasis [4]. In this study, we observed that under external stress, LSPCs displayed a positive response by initiating proliferation and differentiation towards the corneal epithelium. This cellular response aimed to restore homeostasis at the early stage. However, prolonged exposure led to the depletion of LSPCs, resulting in an inability to support corneal homeostatic balance (Fig. 8). This was manifested by a thinner corneal epithelium and the presence of corneal epithelial staining. Additionally, LSPCs also act as a barrier to prevent the conjunctival epithelium from extending onto the cornea [32]. The pterygium originates from dysfunction of LSPCs and manifests as abnormal hyperplasia of the bulbar conjunctiva towards the cornea [33]. Our previous research implied a significant correlation between air pollution and the risk of pterygium development [11]. In this long-term exposure model, we found elevated levels of K10 in the limbus, an increased marker reported in pterygium tissue [34], further confirming the potential of PM2.5 to disrupt the limbal barrier and increase the risk of pterygium.

### PM2.5 exposure and circadian disruption in LSPCs

Moreover, we identified that the circadian rhythm pathway was disturbed in LSPCs exposed to PM2.5. In recent years, a few studies have shown that PM2.5 exposure disrupts circadian rhythms and causes functional and metabolic disorders in different organs [15, 16]. Our results pointed out that core clock genes were dysregulated throughout PM2.5 exposure. While LSPCs behaved differently in the short and long term, suggesting that different degrees of circadian rhythm disturbances have different effects on LSPCs. A study by Li et al. revealed that perturbing the circadian rhythm through sleep deprivation causes LSPCs to proliferate in the short term, while long-term sleep deprivation leads to the loss of LSPCs and early LSCD manifestation [35], which is similar to our results. In the research from Archana et al., the activation of adult neural stem cells in mice is regulated by the day/night cycle and intracellular calcium dynamics [36]. Therefore, short-term PM2.5 exposure activates the proliferation and differentiation of LSPCs by perturbing circadian rhythms; however, long-term circadian rhythm disturbances induced by PM2.5 cause damage and loss of LSPCs, ultimately resulting in LSCD and imbalance of corneal epithelial homeostasis (Fig. 8). Treatments targeting circadian rhythms, such as intermittent fasting or medication at specific times [17], maybe a valuable option for treating PM2.5-associated corneal diseases.

Circadian rhythm disturbances cause mitochondrial respiration alterations, further affecting oxidative phosphorylation [37]. Oxidative phosphorylation and mitochondrial-related processes were also mentioned in our RNA-seq results. Sleep deprivation-induced circadian misalignment can result in an increase in H_2_O_2_ in the tear film of mice [35]. The current study also found elevated H_2_O_2_ in rat tears after short-term exposure. Several studies have revealed that oxidative phosphorylation participates in PM2.5-mediated various diseases [38, 39] and activation of stem cells or progenitors [40, 41]. Thus, circadian rhythm disturbances can affect various pathways and processes, such as oxidative phosphorylation, and further synergistically regulate the activity of LSPCs.

### Limbal microenvironment and circadian disruption in LSPCs

Moreover, prolonged exposure to PM2.5 resulted in the destruction of the limbal microenvironment, as demonstrated by observable structural and functional alterations in the limbal vasculature, decreased corneal nerve density, and accelerated infiltration of immune cells in the limbal stroma. These factors collectively contributed to the development of LSCD. The limbal vasculature has been recognized as a supporter of stemness in LSPCs, and the corneal nerve plays a role in the nutritional supply and self-renewal in LSPCs [6, 42]. In addition, LSPCs lose their stem cell marker under chronic inflammation conditions [43, 44]. Thus, an impaired microenvironment under prolonged exposure eventually leads to the inability to support LSPCs and causes LSCD.

Intriguingly, we found that the early response of the microenvironment to PM2.5 may facilitate the activation of LSPCs. T cells in the limbus have been recognized as niche cells for quiescent LSPCs regulating cell proliferation and wound closures, inhibition of T cells can lead to the proliferation of LSPCs [45]. Notably, there was a decrease in inflammatory cells at early PM2.5 exposure, which might trigger the corresponding activation of LSPCs. Besides, a significantly high level of vascular permeability of limbal vessels was observed as well. In other research, PM2.5 exposure disrupts vessel barriers and enhances vascular permeability, promoting the onset of various diseases [46–49], and these findings are in tandem with ours. However, no study has ever reported whether enhanced vascular leakage is associated with the initiation of LSPCs, and this is a question that deserves further investigation.

Since LSPCs activities are largely supported by their stem cell niche, it is worth discussing whether limbal microenvironment alteration also contributes to the circadian disruption of LSPCs. The immune system is under the control of the circadian rhythm, furthermore, it is demonstrated that immune stimuli such as LPS, IFNα, and TNFα can also affect clock gene expression in multiple cell types and organs [50, 51]. Circadian rhythms are tightly regulated by the neural system as well. Circadian rhythmicity exists in the cholinergic system, the cholinergic anti-inflammatory reflex arc has been implied to regulate the circadian system [52]. Therefore, the change of limbal microenvironment such as the increased immunocytes or the loss of nerves may participate in the circadian rhythm perturbance of LSPCs (Fig. 8), which also needs further exploration.

Finally, we acknowledge that our study has some limitations. PM2.5 exposure affects multiple systems in humans, and our administration of PM2.5 drops on the eye surface of rats does not fully replicate real-world exposure or capture the potential effects on other organs. Nevertheless, this model offers enhanced efficiency in evaluating the direct impact of PM2.5 on ocular surfaces. Secondly, there are limbal stem cells and progenitor cells at different stages of differentiation at the limbus, and further clarification of the effect of PM2.5 on them requires more advanced tools, such as single-cell transcriptome sequencing technology. In addition, the limbal microenvironment is a complex system with multiple cross-regulation between components, which also needs further exploration.

## Conclusion

In conclusion, long-term PM2.5 exposure leads to the destruction of the limbal microenvironment, resulting in LSPC dysfunction and corneal homeostasis disruption, ultimately causing a variety of corneal diseases. Circadian rhythm disturbance is involved in the activation and dysregulation of LSPCs, treatments targeting circadian rhythm can be a potential option to alleviate PM2.5-induced corneal injury.

## Materials and methods

### Clinical data collection and analysis

We collected Fourier-domain Optical Coherence Tomography (FD-OCT) (OPTOVUE, ivue100) corneal pattern images of patients in the outpatient clinic of Zhejiang University Eye Hospital from 2018 to 2022. According to the Chinese Ambient Air Quality Standards (GB3095-2012), the PM2.5 concentration limit for residential areas, mixed commercial and traffic residential areas, cultural areas, general industrial areas, as well as rural areas is 35 µg/m^3^. Therefore, the recruited participants of the current study were divided into 2 groups (< 35 µg/m^3^ and ≥ 35 µg/m^3^) according to the 5-year average annual PM2.5 concentration of their permanent residence regions between 2018 and 2022. Individuals with ocular surface diseases, surgeries or trauma history, and systemic diseases were excluded. Moreover, individuals with a long history of living away from home, smoking, and occupational dust exposure were also excluded. Differences in age composition ratio and male/female ratio between the groups were insignificant.

A previous study conducted by Feng et al. [53] showed that there is no significant difference between the nasal and temporal side of limbal epithelial thickness. Moreover, it was apparent that the LSPCs in nasal and temporal limbus are at a higher risk of PM2.5 exposure than those in the superior and inferior limbus, because the former is not covered by the eyelids. As a result, FD-OCT images of nasal or temporal limbus were collected from individuals in all groups for further analysis in this study. Limbal epithelium thickness was manually measured as reported by the previous study [31]. Briefly, FD-OCT images of the limbus were imported into the IPP6.0 software. The anterior and posterior margins of the limbal epithelium were defined as follows: The anterior margin is the line connecting the terminals of the Bowman’s and Descemet’s membranes, while the posterior margin is a vertical line from the scleral spur to the surface of the eye, intersecting the outer surface of the eye, the whole length was approximately 1 mm (Fig. 1A). The average, maximum and minimum values of corneal epithelial thickness were measured by calculating the distance between the upper and lower margins by IPP6.0 software. The measurement value for one eye was used in subsequent analysis.

### PM2.5 extract preparation

The PM2.5 extraction process was as previously reported [13]. Briefly, atmospheric PM2.5 samples were collected using quartz filters, soaked, and sonicated in 75% ethanol for 30 min to obtain PM2.5 suspensions. After lyophilization, the PM2.5 samples were resuspended and diluted to 5 mg/mL in phosphate-buffered saline (PBS, 1×) as the storage medium, and stored at -80°C for subsequent assays. The PM2.5 samples were collected at 1.5 m above ground, which corresponds to the respiratory zone height, in the yard of the Center for Disease Control and Prevention of Zhejiang Province, Hangzhou, Zhejiang, China. Lyophilized PM2.5 sample analysis, including organic, ion, and metal components was as previously described [13].

### Animal experiments

#### Establishment of PM2.5-exposed rat models

 Male Sprague-Dawley rats (8 weeks old, 200–300 g in weight) were obtained from SLAC Laboratory Animal Co., Ltd. (Shanghai, China), housed in a standard environment with a temperature of 20 to 24 °C, a humidity of 50 to 60% under a 12-hour light-dark cycle and provided with food and water without limitation. Rats were randomized into 4 groups. For the long-term PM2.5 exposure group, both eyes of rats were administered with 5 µL eye drops of PM2.5 (1 mg/mL in PBS) for 21 days at a frequency of 4 times per day. The actual daily dose was 20 µg. The rationale for the exposure treatment in rat models was described in **Text. S1**. For the short-term PM2.5 exposure group, both eyes of rats were administered with 5 µL eye drops of PM2.5 (1 mg/mL in PBS) for 2 days at the same frequency. Rats that were administered with eye drops of PBS for the same days were established as control groups. N = 35 in each group of long-term models. N = 20 in each group of short-term models.

#### Ocular surface fluorescein sodium staining

 Rat corneal epithelium defects were explored after 0, 2, 7, 14, and 21 days of administration using the Fluorescein sodium strips (Meizilin). After anesthesia by intraperitoneal injection of 0.3% pentobarbital sodium (50 mg/kg), fluorescein sodium strips were moistened with PBS and placed in conjunctival sacs of the lower lids of rats. The eyes of the rats were gently closed several times and the staining of the ocular surface was examined with a slit lamp under cobalt blue light.

#### Tear secretion test and tear H_2_O_2_ measurement

To evaluate tear film stability and ocular surface damage, tear secretion was measured after 0, 2, 7, 14, and 21 days of administration using Schirmer’s test. After anesthesia, a graded sterile phenol red cotton thread for a tear test (Meizilin) was gently placed in the conjunctival sac of the lower lid. Rat eyes were closed for 60 s and the length of the wetting thread was measured in millimeters.

H_2_O_2_ in rat tears was measured after 0, 2, 7, 14, and 21 days of administration. After anesthesia, 10 µl of PBS was applied to the ipsilateral eye of all rats, and recovered after wetting the eye surface. The level of H_2_O_2_ was measured using H_2_O_2_ Detection Kit (mlbio#m1076343).

#### Vessel leakage detection

Vessel leakage of limbal vessel after 0, 2, 7, 14, and 21 days of administration was measured using Evans blue. Evans blue (30 mg/kg; Sigma-Aldrich #E2129) was injected via rat tail veins(n = 5), circulated for 2 h, and final blood samples for each rat were obtained. After anesthesia and opening the chest cavity, rats were perfused with 37 °C PBS via the left ventricle until the color of the fur lightened and the liquid flowing from the right atrium was clean. Both eyes were immediately enucleated and bisected at the equator, after which the corneas with limbus were dissected under a microscope. The corneas were dried at 4 °C overnight and cornea dry weights were measured. The cornea specimen was incubated in 0.2 ml formamide (Macklin# F809511) at 70 °C overnight to extract the Evans blue dye. The extract and blood samples were centrifuged at 17,000 g for 15 min and 60 µl of the supernatant was used to measure absorbance at 630 nm. A standard curve of Evans blue in formamide was established to calculate the concentrations of dyes. Vessel leakage levels were calculated using the following formula:

$$\begin{array}{l}{\rm{Evans\ blue (}}\mu g) \times \\{\rm{Circulation\ time (h)/}}\\{\rm{[Cornea\ dry\ weight (g)}}\\\times {\rm{Plasma\ Evans }}\\{\rm{blue\ concentration (}}\mu g/\mu l)]\end{array}$$

### Sample collection

For limbal epithelium RNA sequencing, rats were sacrificed after 2 days of administration. Eyeballs were collected(n = 3) and rinsed by PBS containing 50 µg/mL gentamicin and 1.25 µg/mL amphotericin B. Limbal segments were separated from eyeballs using ophthalmic scissors and the remaining conjunctiva, iris, and corneal endothelium was removed. The intact limbal epithelium sheet from the limbal segment was isolated by digestion at 37 °C for 20 min with 10 mg/ml Dispase II (Sigma-Aldrich, #D4693) in a modified embryonic stem cell medium made of 1:1 mixture of Dulbecco’s modified Eagle’s medium and Ham’s F12 medium containing 5 ng/ml epidermal growth factor, 5 µg/ml insulin, 5 µg/ml transferrin, 5 ng/ml sodium selenite, 0.5 µg/ml hydrocortisone, 30 ng/ml cholera toxin B, 50 µg/ml gentamicin, 1.25 µg/ml amphotericin B and 10% fetal bovine serum.

For H&E staining and immunofluorescence staining, rats were sacrificed after 2 or 21 days of administration. Eyeballs were obtained and directly embedded in optimum cutting temperature compound (OCT) in liquid nitrogen and sectioned to 8 μm using a Leica CM1950. Extra eyeballs were stored in liquid nitrogen for a long time.

### H&E staining and epithelium thickness measurement in rats

Sections were fixed, incubated with Hematoxylin, washed, stained with Eosin, dehydrated in 95% and 100% Ethanol, incubated with Xylene, and mounted.

For the measurement of the cornea, we measured the average epithelial thickness within 400 microns of the central cornea using IPP6.0 software. For the measurement of the limbus, we examined the thickness of the limbal epithelium located at the transition zone of the corneal epithelium and conjunctival epithelium. The maximum values of the distance between the upper and lower margins of the limbal epithelium were calculated by IPP6.0 software.

### Immunofluorescence staining

The rat cornea sections were soaked in PBS for 10 min to remove OCT and fixed with 4% paraformaldehyde for 15 min. After incubation with 10% goat serum and 0.2% TritonX-100 (Sigma Aldrich) for 1 h at room temperature, samples were incubated with primary antibodies at 4℃ overnight. The next day, they were incubated with secondary antibodies at room temperature for 1 h, labeled with 2-(4-Amidinophenyl)-6-indolecarbamidine dihydrochloride (Sigma Aldrich), and covered with coverslips. Observation and imaging of samples were performed using the Leica TCS SP8 confocal microscope (Leica, Wetzla, Germany). The antibodies used in this study were: Ki67 (1:400, Cell Signaling Technology #9129s), K12 (1:200, Santa Cruz Biotechnology #sc-515,882), K10 (1:200, Abcam #ab76318), and CD45 (1:150, Abcam #ab10558).

Whole-mount immunofluorescence examination was performed to assess vascular and nerve morphologies, and tight junction. Rat corneas containing limbus were dissected from the eyeballs’ posterior aspect of the corneoscleral rim using fine scissors. After removing the iris and retina, corneas were fixed in 4% paraformaldehyde on ice for 1 h, followed by permeabilization and blocking for 1 h. Cornea specimens were incubated with primary antibodies at 4℃ overnight, and then probed with secondary antibodies for 1 h at room temperature and nuclei staining the next day. Anti-rabbit CD31 antibody (1:100, Abcam#ab222783) was used for vascular staining, and anti-rabbit ZO-1 antibody (1:400, Proteintech# 21773-1-AP) was used for tight junction staining. For cornea nerve staining, corneas were incubated with NorthernLights™ NL557-conjugated mouse monoclonal anti-TUBB3 (1:10, R&D#NL1195R) at 4℃ overnight, and their nuclei were stained. The cornea was cut into 4 flaps, the endothelial side was turned upwards and covered with coverslips. The Leica TCS SP8 confocal microscope (Leica) was used to observe and image the corneal whole mount splices. The density of vascular and neural networks and the mean fluorescence intensity of tight junction were analyzed by Image J2 v2.3.0.

### RNA-seq of limbal epithelium sheets

The total RNA of limbal epithelium sheets was extracted by FlaPure Animal Tissue Total RNA Extraction Kit (Genesand# RE705). Nanodrop ND-1000 and Agilent 5400 were used to measure the concentration, integrity, and purity of RNA, and qualified samples were used for the subsequent RNA-seq. RNA-seq was performed by Novogene Co., LTD (Beijing, China).

The protocols used were adopted from our previous work [14]. Briefly, NEBNext® UltraTM RNA Library Prep Kit for Illumina® (NEB, Beverly, MA, USA) was used to prepare sequencing cDNA libraries. To obtain clean data, reads containing ploy-N and low-quality reads needed to be removed from raw data. DESeq2 R package was used for differential expression analysis. The DEGs were determined based on the adjusted *p*-value < 0.001 and | log2(fold change) | > 0.7. GO enrichment analysis and KEGG pathways were implemented by the clusterProfiler R package, and terms with corrected *p*-value < 0.05 were considered significantly enriched by the DEGs. PPI network among DEGs was analyzed on the website of STRING (https://cn.string-db.org/).

### RNA extraction, reverse transcription, and qPCR

After isolation of total RNA of limbal epithelium, reverse transcription was performed using PrimeScript™ RT Master Mix Kit (Takara, # RR036), and Synthesized cDNA was amplified in duplicate on an ABI Prism 7500 Fast Sequence Detection System (Thermo Fisher Scientific) using TB Green® Premix Ex Taq™ II (Takara, #RR420). Samples were denatured at 95 °C for 30s, followed by 40 cycles at 95 °C for 3s, 60 °C for 30s. The primers used were listed in Table S3.

### Statistical analysis

Data are expressed as mean ± standard deviation and were analyzed using GraphPad Prism 9 (GraphPad Software, San Diego, CA) and SPSS version 26 (SPSS, Inc., Chicago, IL, USA). The unpaired *t*-test was used to compare the mean values between two groups, while the means for multiple groups were compared by one-way ANOVA. Spearman’s correlation analysis was used to assess the association between PM2.5 concentrations and limbal epithelial thickness. *p* < 0.05 was the threshold for statistical significance.

### Electronic supplementary material

Below is the link to the electronic supplementary material.

**Additional file 1: Table S1.** Mean/maximum/minimum value of limbal epithelium thickness in the two groups. *p < 0.05, **p < 0.01.

**Additional file 2: Table S2** Detail of Spearman analysis table. *p < 0.05.

**Additional file 3: Table S3** The primers used for qPCR.

**Additional file 4: Supplemental figures Fig. S1.** Meta-analysis of fine particulate matter on corneal diseases. This meta-analysis was divided into the following two parts according to the data types in the study (binary variables or continuous variables). **(A)** 6 studies were plotted in a forest of binary variables and odds ratios (OR) were calculated (OR [95%CI]: 1.13 [1.05, 1.21], p = 0.001; Heterogeneity: I2 = 95%, p = 0.000. The heterogeneity exists). **(B)** 5 studies were plotted in a forest of continuous variables and regression coefficients (β) were calculated (β [95%CI]: 0.51 [0.38, 0.63], p = 0.000; Heterogeneity: I2 = 98.4%, p = 0.000. The heterogeneity exists). The final merged results indicated that fine particulate matter exposure is positively associated with corneal disease or related symptoms. **Fig. S2.** Ocular surface fluorescein sodium staining in short-term PM2.5 exposure rat model by slit lamp examination (N = 6 per group). **Fig. S3.** Schirmer’s test revealed that tear secretion was not notably affected by PM2.5 exposure after 2 days (N = 6 per group). **Fig. S4.** Limbal vascular morphology of short-term PM2.5 exposure rat model (scale bar, 300 μm) (N = 3 in each group). **Fig. S5.** Corneal innervation of short-term PM2.5 exposure rat model (scale bar, 100 μm) (N = 3 in each group).

**Additional file 5: text. S1** The rationale for the exposure treatment rat models.

## Data Availability

All data generated or analyzed during this study are included in this published article [and its supplementary information files].

## Abbreviations

LSPCs: Limbal stem/progenitor cells
ECM: Extracellular matrix
LSCD: Limbal stem cell deficiency
K12: Keratin12
K10: Keratin10
FD-OCT: Fourier-domain Optical Coherence Tomography
PM2.5: Fine particulate matter less than 2.5 microns in diameter
RNA-seq: RNA-sequencing
GO: Gene Ontology
KEGG: Kyoto encyclopedia of genes and genomes
DEGs: Differentially expressed genes
BP: Biological process
MF: Molecular function
CC: Cellular component
qPCR: quantitative fluorescent polymerase chain reaction
PPI: Protein-protein interaction
H_2_O_2_: hydrogen peroxide
OCT: optimum cutting temperature compound
