# Supplementary material for: Long-term PM2.5 exposure disrupts corneal epithelial homeostasis by impairing limbal stem/progenitor cells in humans and rat models
Source: Part Fibre Toxicol. 2023 Sep 27;20:36. doi: 10.1186/s12989-023-00540-y (PMC10523760; doi:10.1186/s12989-023-00540-y)
Supplement: Supplementary file 4 — Additional file 4: Supplemental figures Fig. S1. Meta-analysis of fine particulate matter on corneal diseases. This meta-analysis was divided into the following two parts according to the data types in the study (binary variables or continuous variables). (A) 6 studies were plotted in a forest of binary variables and odds ratios (OR) were calculated (OR [95%CI]: 1.13 [1.05, 1.21], p = 0.001; Heterogeneity: I2 = 95%, p = 0.000. The heterogeneity exists). (B) 5 studies were plotted in a forest of continuous variables and regression coefficients (β) were calculated (β [95%CI]: 0.51 [0.38, 0.63], p = 0.000; Heterogeneity: I2 = 98.4%, p = 0.000. The heterogeneity exists). The final merged results indicated that fine particulate matter exposure is positively associated with corneal disease or related symptoms. Fig. S2. Ocular surface fluorescein sodium staining in short-term PM2.5 exposure rat model by slit lamp examination (N = 6 per group). Fig. S3. Schirmer’s test revealed that tear secretion was not notably affected by PM2.5 exposure after 2 days (N = 6 per group). Fig. S4. Limbal vascular morphology of short-term PM2.5 exposure rat model (scale bar, 300 μm) (N = 3 in each group). Fig. S5. Corneal innervation of short-term PM2.5 exposure rat model (scale bar, 100 μm) (N = 3 in each group). [file 12989_2023_540_MOESM4_ESM.docx]

**Long-term PM2.5 exposure disrupts corneal epithelial homeostasis by impairing limbal stem/progenitor cells in humans and rat models**

Shengjie Hao^1#^, Zhijian Chen^2#^_,_ Yuzhou Gu^1#^, Lu Chen ^1#^, Feiyin Sheng^1^, Yili Xu^1^, Di Wu^1^, Yu Han^1^, Bing Lu^1^, Shuying Chen^1^, Wei Zhao^1^, Houfa Yin^1^, Xiaofeng Wang^2^, S Amer Riazuddin^3^, Xiaoming Lou^2*^, Qiuli Fu^1*^, Ke Yao^1*^

^1^ Eye Center of the 2nd Affiliated Hospital, School of Medicine, Zhejiang University, Zhejiang Provincial Key Lab of Ophthalmology, Hangzhou, Zhejiang Province, China

^2^ Department of Environmental and Occupational Health, Zhejiang Provincial Center for Disease Control and Prevention, Hangzhou, Zhejiang Province, China

^3^ The Wilmer Eye Institute, Johns Hopkins University School of Medicine, Baltimore, USA

***Correspondence:** Ke Yao, MD., Eye Center of the 2nd Affiliated Hospital, Medical College of Zhejiang University, Hangzhou 310009, Zhejiang Province, China; [xlren@zju.edu.cn](mailto:xlren@zju.edu.cn).

***Co-correspondence:** Qiuli Fu, Ph.D., Eye Center of the 2nd Affiliated Hospital, Medical College of Zhejiang University, Hangzhou 310009, Zhejiang Province, China; [2313009@zju.edu.cn](mailto:2313009@zju.edu.cn).

***Co-correspondence:** Xiaoming Lou, Department of Environmental and Occupational Health, Zhejiang Provincial Center for Disease Control and Prevention, Hangzhou 310051, Zhejiang Province, China; [xmlou@cdc.zj.cn](mailto:xmlou@cdc.zj.cn).

^#^ S.H., Z.C., Y.G. and L.C. contributed equally to this work.

**Supporting Figures**

**Table of Contents**

**Fig.S1.** Meta-analysis of fine particulate matter on corneal diseases.

**Fig.S2.** Ocular surface fluorescein sodium staining in short-term PM2.5 exposure rat model by slit lamp examination.

**Fig.S3.** Schirmer’s test revealed that tear secretion was not notably affected by PM2.5 exposure after 2 days.

**Fig.S4.** Limbal vascular morphology of short-term PM2.5 exposure rat model (scale bar, 300µm).

**Fig.S5.** Corneal innervation of short-term PM2.5 exposure rat model (scale bar, 100µm).


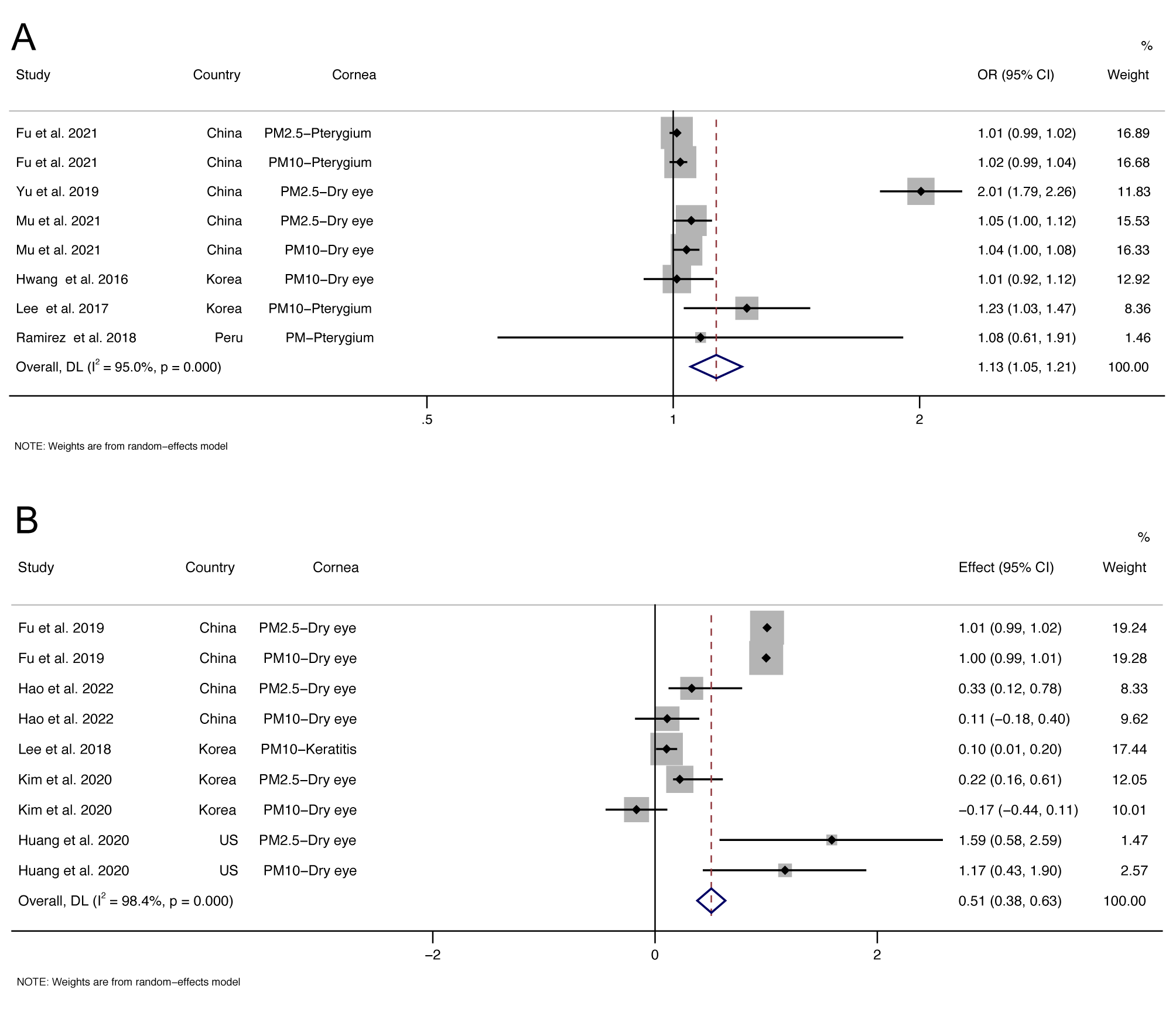


**Fig.S1. Meta-analysis of fine particulate matter on corneal diseases.** The meta-analysis was divided into the following two parts according to the data types in the study (binary variables or continuous variables). **(A)** 6 studies were plotted in a forest of binary variables and odds ratios (OR) were calculated (OR [95%CI]: 1.13 [1.05, 1.21], p=0.001; Heterogeneity: I2=95%, p=0.000. The heterogeneity exists). **(B)** 5 studies were plotted in a forest of continuous variables and regression coefficients (β) were calculated (β [95%CI]: 0.51 [0.38, 0.63], p=0.000; Heterogeneity: I2=98.4%, p=0.000. The heterogeneity exists). The final merged results indicated that fine particulate matter exposure is positively associated with corneal disease or related symptoms.


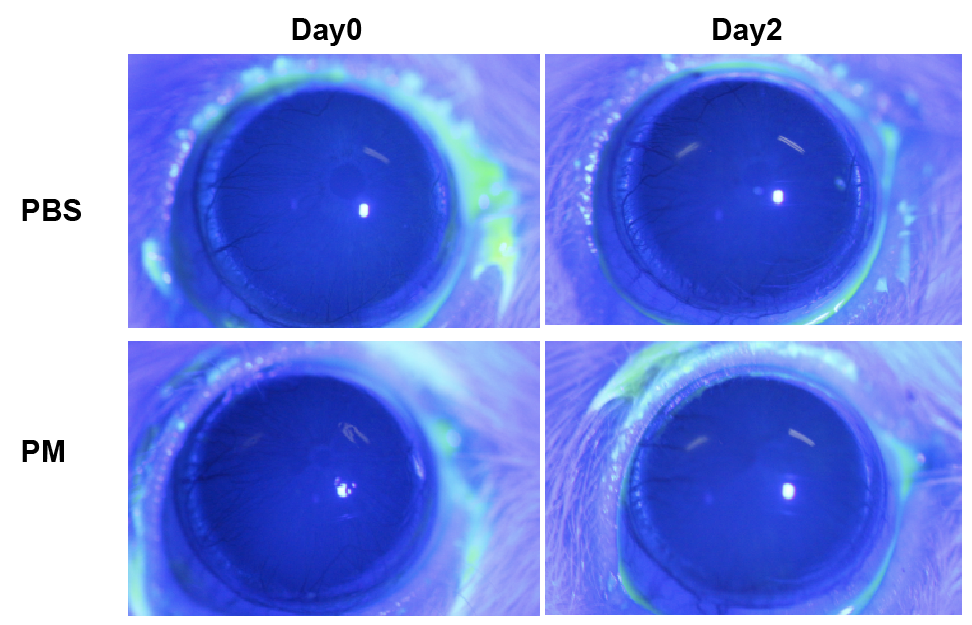


**Fig.S2.** Ocular surface fluorescein sodium staining in short-term PM2.5 exposure rat model by slit lamp examination (n=6 per group).

**Fig.S3.** Schirmer’s test revealed that tear secretion was not notably affected by PM2.5 exposure after 2 days (n=6 per group).


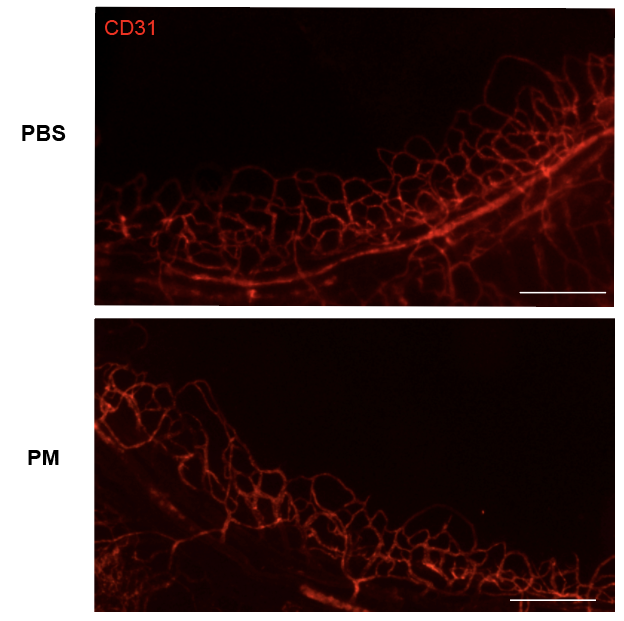


**Fig.S4.** Limbal vascular morphology of short-term PM2.5 exposure rat model (scale bar, 300µm). N=3 in each group.


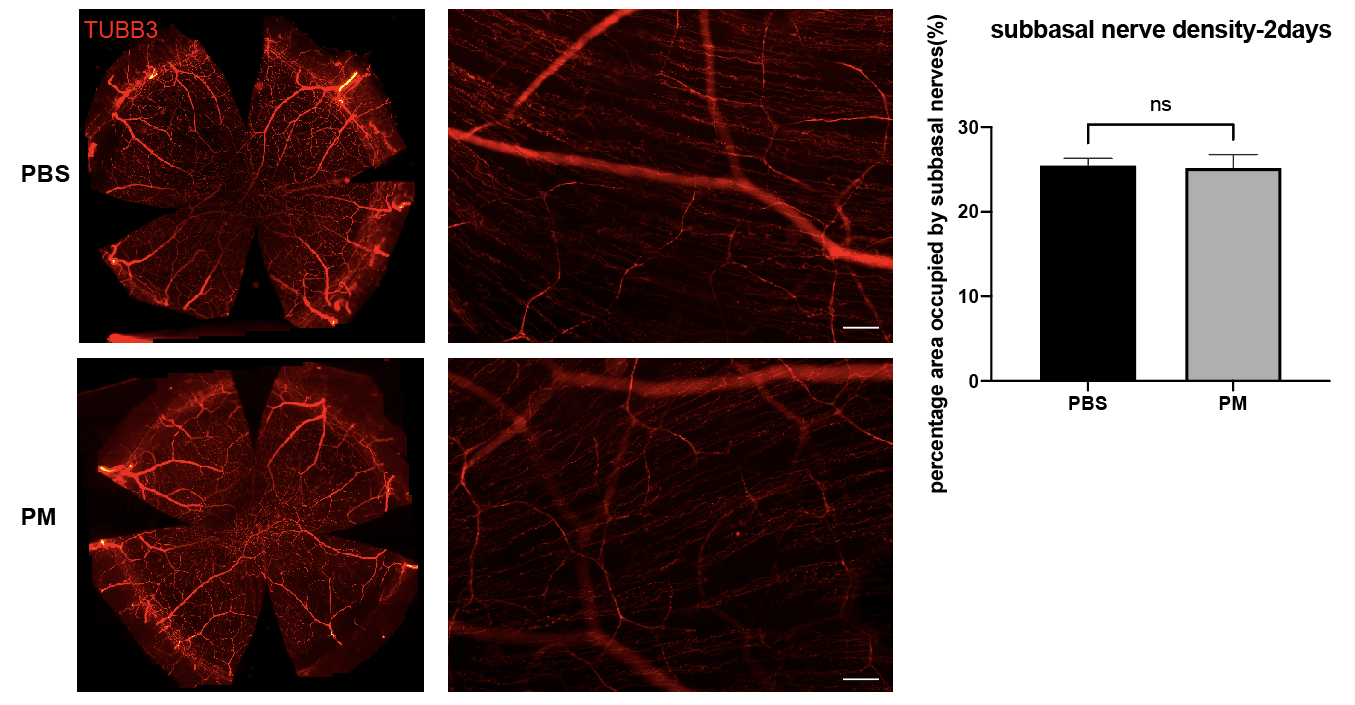


**Fig.S5.** Corneal innervation of short-term PM2.5 exposure rat model (scale bar, 100µm). N=3 in each group.
