# Supplementary material for: Long-term PM2.5 exposure disrupts corneal epithelial homeostasis by impairing limbal stem/progenitor cells in humans and rat models
Source: Part Fibre Toxicol. 2023 Sep 27;20:36. doi: 10.1186/s12989-023-00540-y (PMC10523760; doi:10.1186/s12989-023-00540-y)
Supplement: Supplementary file 5 — Additional file 5: text. S1 The rationale for the exposure treatment rat models. [file 12989_2023_540_MOESM5_ESM.docx]

**Long-term PM2.5 exposure disrupts corneal epithelial homeostasis by impairing limbal stem/progenitor cells in humans and rat models**

Shengjie Hao^1#^, Zhijian Chen^2#^_,_ Yuzhou Gu^1#^, Lu Chen ^1#^, Feiyin Sheng^1^, Yili Xu^1^, Di Wu^1^, Yu Han^1^, Bing Lu^1^, Shuying Chen^1^, Wei Zhao^1^, Houfa Yin^1^, Xiaofeng Wang^2^, S Amer Riazuddin^3^, Xiaoming Lou^2*^, Qiuli Fu^1*^, Ke Yao^1*^

^1^ Eye Center of the 2nd Affiliated Hospital, School of Medicine, Zhejiang University, Zhejiang Provincial Key Lab of Ophthalmology, Hangzhou, Zhejiang Province, China

^2^ Department of Environmental and Occupational Health, Zhejiang Provincial Center for Disease Control and Prevention, Hangzhou, Zhejiang Province, China

^3^ The Wilmer Eye Institute, Johns Hopkins University School of Medicine, Baltimore, USA

***Correspondence:** Ke Yao, MD., Eye Center of the 2nd Affiliated Hospital, Medical College of Zhejiang University, Hangzhou 310009, Zhejiang Province, China; [xlren@zju.edu.cn](mailto:xlren@zju.edu.cn).

***Co-correspondence:** Qiuli Fu, Ph.D., Eye Center of the 2nd Affiliated Hospital, Medical College of Zhejiang University, Hangzhou 310009, Zhejiang Province, China; [2313009@zju.edu.cn](mailto:2313009@zju.edu.cn).

***Co-correspondence:** Xiaoming Lou, Department of Environmental and Occupational Health, Zhejiang Provincial Center for Disease Control and Prevention, Hangzhou 310051, Zhejiang Province, China; [xmlou@cdc.zj.cn](mailto:xmlou@cdc.zj.cn).

^#^ S.H., Z.C., Y.G. and L.C. contributed equally to this work.

**Text S1: The rationale for the exposure treatment in rat models**

According to the data provided by the National Ministry of Environmental Protection of China and Environmental Protection Agency of US, 100~250 μg/m^3^ was chosen as the PM2.5 concentration for mild PM2.5 pollution. As an example, the estimated time spent outdoors per day was set at 2 hours (7200 seconds) for an adult engaged in indoor work, the average radius of the adult cornea was about 6 mm, and the movement speed of a lightly active adult was estimated to be 1 m/s. The mass of daily PM2.5 exposure is then calculated as follows:

mass of daily PM2.5 exposure = V ∙ c = S ∙ v ∙ t ∙ c

= π × (0.006 m)^2^ × 1m/s × 7200 s × (100~250) μg/m^3^

≈ (80~200) μg

V - volume of air in contact with one cornea per hour

c - the concentration of PM2.5 in the air

S - surface area of the cornea

v - the speed of movement of a person during light activity

t - exposure time of PM2.5

m - meter

s - second

m^3^ - cubic meter

Based on this estimation, considering that the average radius of the rat cornea is about 3 mm, the equivalent exposure mass for rats should be 20-50 μg. In this study, the volume of each eye drop was 5 μL, the daily PM2.5 exposure mass in a rat is calculated as follows: 1 mg/mL × 5 μL ×4 times/day = 20 μg.
